# A genome-wide tree- and forest-based association analysis of comorbidity of alcoholism and smoking

**DOI:** 10.1186/1471-2156-6-S1-S135

**Published:** 2005-12-30

**Authors:** Yuanqing Ye, Xiaoyun Zhong, Heping Zhang

**Affiliations:** 1 Department of Epidemiology and Public Health, Yale University School of Medicine, New Haven, CT 06520-8034 USA

## Abstract

Genetic mechanisms underlying alcoholism are complex. Understanding the etiology of alcohol dependence and its comorbid conditions such as smoking is important because of the significant health concerns. In this report, we describe a method based on classification trees and deterministic forests for association studies to perform a genome-wide joint association analysis of alcoholism and smoking. This approach is used to analyze the single-nucleotide polymorphism data from the Collaborative Study on the Genetics of Alcoholism in the Genetic Analysis Workshop 14. Our analysis reaffirmed the importance of sex difference in alcoholism. Our analysis also identified genes that were reported in other studies of alcoholism and identified new genes or single-nucleotide polymorphisms that can be useful candidates for future studies.

## Background

Alcoholism is a complex disease that is highly concordant within family clusters. It is a widespread problem; nearly 14 million Americans abuse alcohol or are alcoholic [1]. It is a major cause of certain cancers, especially liver cancer, a risk factor for brain damage, and is hazardous for developing fetuses. The Genetic etiology of alcoholism is well documented but not well understood [2], though the results of controlled family and twin studies of alcoholism suggest that alcoholism is in part caused by genetic components [3].

Smoking is highly associated with alcohol dependence [4]. Genetic factors contribute to a person's risk of both smoking and alcoholism [4]. There is a high prevalence of smoking among active alcoholics. The analysis of a 1981 Australian twin panel cohort data finds a positive genetic correlation between habitual smoking and alcoholism [5]. The effect remains significant even after controlling for personality variables. Thus, the joint analysis of alcohol dependence and smoking using genetic information should reveal interesting results.

Classification trees and forests are known for their ability to identify complex relationships, especially in large, complex datasets [6]. The availability of the single-nucleotide polymorphism (SNP) data in the Collaborative Study on the Genetics of Alcoholism (COGA) makes these methods well suited for identifying SNPs associated with smoking and alcoholism. In fact, we identified multiple trees of similar quality in terms of prediction error, and those trees suggest multiple potential genetic pathways underlying smoking and alcoholism.

## Methods

### Data structure

The COGA data include 1,614 family members. After removing those individuals with missing genotype data on some markers, there were 1,306 individuals in the Illumina genotype dataset. There are 4,752 SNP markers released by Illumina, 32 of them without a map position. The number of SNPs released in the reformatted data was 4,720. Phenotypes used for this analysis are alcohol dependence based on DSM-III-R and Feighner, coded as ALDX1, and smoking. We combined ALDX1 with smoking to construct a comorbid response. Because ALDX1 has 4 levels (261 pure unaffected, 28 never drank, 408 unaffected with some symptoms, 609 affected), the comorbid response has 8 levels. The covariates include sex, parental phenotypes, and the SNP markers. The inclusion of parental phenotypes in such an association analysis is well documented to control for the residual familial correlations [7]. The coding scheme for a SNP genotype is 0 for 1/1, 1 for 1/2, and 2 for 2/2. A variable, sex, was used to account for any sex differences.

### Classification trees

**Figure 1:**
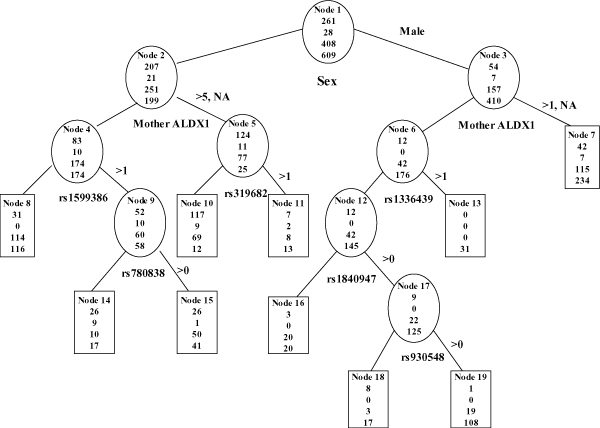
**The pruned tree at the significance level of 0.00001 for ALDX1 using Illumina SNP data. **We use circles and boxes to represent internal and terminal nodes, respectively. Under each internal node is the covariate that is used to split the node. Inside each node and from top down are the node number, the numbers of pure unaffected individuals, never drink individuals, unaffected individuals with some symptom, and affected individuals.

**Figure 2:**
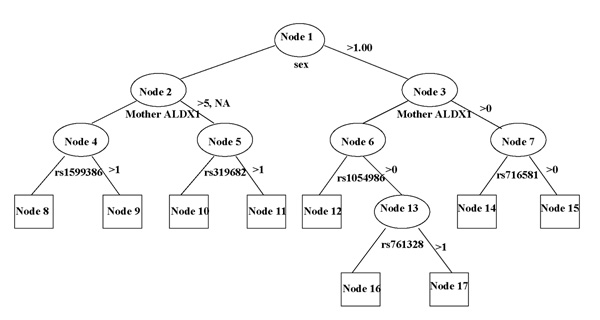
**The pruned tree at the significance level of 0.0001 for comorbid ALDX1 and smoking using Illumina SNP data. **We use circles and boxes to represent internal and terminal nodes, respectively. Under each internal node is the covariate that is used to split the node.

The tree construction consists of two steps: tree growing and pruning. Tree growing is based on recursive partitioning. The classification tree for ALDX1 as the single outcome is shown in Figure 1, while Figure 2 depicts the classification tree for comorbid ALDX1 and smoking.

In Figure 1, the root node at the top contains all study samples. We use circles and boxes to represent internal nodes and terminal nodes, respectively. A splitting rule consists of a covariate and its corresponding threshold. As shown in Figure 1, sex is selected to split the root node with males to the right daughter node and females to the left daughter node, underscoring prominent sex difference. The selection of such a split is based on a specific goodness of split measure such as entropy [6]. The objective of the split is to produce two daughter nodes (numbers 2 and 3 in Figure 1) such that the within-node distribution of the phenotype such as ALDX1 in Figure 1, is as homogeneous as possible. Specifically, suppose that we consider splitting node *t*, which can be the root node, and that the outcome variable has *q *levels, which is 4 for ALDX1 and 8 for the combination of ALDX1 and smoking. The entropy-based goodness of split is defined as



where *t*_*L *_and *t*_*R *_are left and right daughter nodes of node *t *resulting from split *s*, respectively,  is the probability for an individual to be in node *t*_*L*_,  is the probability for an individual in node *t*_*L *_to have response level *i*(*i *= 1, ..., *q*). The definitions for  and  are analogous to those of  and . The split based on the sex variable for the root node in Figure 1 was selected because it yielded the highest *i*(*s*) after evaluating all possible splits of the root node using all covariates and all SNPs.

After splitting the root node into two daughter nodes, we repeated the procedure to further partition the daughter nodes into the next layer, and as a result, the study sample is divided into smaller, and hopefully more homogeneous, daughter nodes hierarchically or recursively. This recursive partitioning procedure produces an initial tree that usually contains many nodes. Because there are a finite number of ways of splitting any given study sample, the recursive partitioning can run for a while, but always terminates when it exhausts all possible splits. To improve the reliability and interpretability of the information contained in a tree, the initial tree from the recursive partitioning procedure is usually pruned to a smaller size.

We adopted the bottom-up method described in Zhang and Singer [6] to delete those superficial or unreliable splits. A *χ*^2 ^testing statistic for a 2 × *q *contingency table was calculated for each internal node. For example, in Figure 1, we have the 2 × 4 table as shown in Table 1 for the root node and the *χ*^2 ^value equals 189.8 for testing the independence of cell counts in the table. After the *χ*^2 ^values are obtained for all internal nodes, we can follow the suggestion of [6] by prespecifying a significance level (e.g., 0.01) and void all splits whose *χ*^2 ^values as well as the *χ*^2 ^values in the subsequent splits do not exceed the predetermined threshold. This pruning step resulted in the tree in Figure 1 for ALDX1.

### Deterministic forest

Thanks to a large number of covariates, we may have multiple splits with similar quality in terms of the goodness of split measure and the predictive precision of the phenotype. Biologically, it is possible that there are multiple pathways to a disease. Thus, it is useful to unravel and make use of all competitive split, and form a forest of competitive trees. Although random forests [8] provide a popular option, for the reasons explained in [9], we adopted the approach in [9] to form a deterministic forest. The key points made in [9] are that the deterministic forests perform similarly to random forests for data similar the COGA data and that the deterministic forests are reproducible and can be studied easily, whereas random forests are produced with uncertainty by design that may be not desirable. We refer to [9] for further discussions.

Following the recommendation in [9], we consider the top 20 splits of the root node and the top 3 splits of the two daughter nodes of the root node, giving rise to a maximum of 180 (20 × 3 × 3) trees in the forest.

## Results

Using the method described above, we obtained an initial tree with 139 nodes for ALDX1. At the significance level of 0.0001 based on a 2 × 4 contingency table, a tree with 39 nodes is determined. At significance level of 0.00001, a tree with 19 nodes is selected as shown in Figure 1. Figure 1 identified six important SNP markers that appear to be significantly associated with alcoholism. We list the SNP markers that are selected when ALDX1 or ALDX1 and smoking are used as the responses in Table 2.

## Discussion

In this report, we identified 37 SNPs that are associated with alcoholism and smoking. Fifteen of these SNPs are within known genes. Table 3 lists the eight genes with known or inferred functions. For example, SNP marker rs476646 is from gene SLC6A13, i.e., member 13 in the solute carrier family 6 (neurotransmitter transporter, GABA) in the chromosome region 12p13. GABA is neurotransmitter in the human central nervous system as well as human liver. Evidence indicates that GABA genes are likely candidates for alcohol dependence, and increased clearance of GABA by the liver is susceptible to alcoholism. It is not surprising that the transporter of these genes is associated to the alcohol addiction [10]. According to our MedLine search, the remaining SNPs and the corresponding genes that we identified have not been previously suggested to be specifically associated with either alcoholism or smoking. However, in a recent genome-wide scan for smoking genes [11], strong or suggestive evidence for linkage on chromosomes 9, 11, 14, and X was reported. While that scan [11] identified the genes on chromosomes 9, 11, and 14 in different regions from what we identified, the SNPs (rs1934176, rs1536163, rs2015312, rs204141, and rs204165) that we identified on the X chromosome are in the same regions as those identified by Gelernter et al. [11]. It is noteworthy that our analysis supports the strong sex difference in alcoholism, which is well documented. For example, Zhang and Merikangas [12] suggested the need to use a lower threshold of alcoholism for females. This is another important motivation for us to analyze the ordinal spectrum of the alcoholism, and may explain partially why most of the SNPs that we have identified were not previously identified to be associated or linked to alcoholism or smoking.

## Abbreviations

COGA: Collaborative Study on the Genetics of Alcoholism

SNP: Single-nucleotide polymorphism

## Figures and Tables

**Table 1:** The 2 × 4 Table for root node

Response levels	Node 2	Node 3
1	207	54
2	21	7
3	251	157
4	199	401

**Table 2:** The identified SNPs

SNP label	Trait^a^	Chromosome
rs930548	A	1
rs628667	A	1
rs1338221	A	1
rs1840947	A	2
rs1516003	C	2
rs986909	A	3
rs1599386	A,C	3
rs319682	A	3
rs728937	A	5
rs1325182	A	6
rs234	A	7
rs940864	C	7
rs1054879	C	9
rs886017	C	9
rs913258	C	9
rs780838	A	10
rs1336439	A	10
rs869451	C	11
rs1149014	A	12
rs1165678	A	12
rs476646	C	12
rs296736	C	12
rs14067	A,C	13
rs759364	A,C	14
rs1972603	A	18
rs1380148	A	22
rs1037193	A	X
rs1349846	A	X
rs1402076	A	X
rs1656651	A,C	X
rs1921708	A,C	X
rs1934176	A	X
rs966446	A,C	X
rs1536163	C	X
rs2015312	C	X
rs204141	C	X
rs204165	C	X

^a^A, the ones significant for ALDX1 only; C, for comorbidity ALDX1 and smoking

**Table 3:** SNPs within known genes

SNP marker	Gene	Chromosome region
rs930548	KCND3	1p13
rs940864	CLCN1	7q35
rs886017	RALGDS	9q34
rs476646	SLC6A13	12p13
rs319682	MAP4	3q21
rs1054879	FREQ	9q33~9q34
rs780838	CUBN	10p12
rs1349846	IL1RAPL1	Xp22 ~ Xp21

^a^A, the ones significant for ALDX1 only; C, for comorbidity ALDX1 and smoking
